# Optimal administration time of vitamin C after ^131^I therapy in differentiated thyroid cancer based on propensity score matching

**DOI:** 10.3389/fsurg.2022.993712

**Published:** 2022-09-22

**Authors:** Ye Liu, Yuhua Wang, Wanchun Zhang

**Affiliations:** ^1^ Department of Nuclear Medicine, Shanxi Bethune Hospital, Shanxi Academy of Medical Sciences, Tongji Shanxi Hospital, Third Hospital of Shanxi Medical University, Taiyuan, China; ^2^ Tongji Hospital, Tongji Medical College, Huazhong University of Science and Technology, Wuhan, China

**Keywords:** radioiodine therapy, salivary glands, sialadenitis, thyroid cancer, vitamin c

## Abstract

**Objectives:**

This study aimed to investigate the protection of the salivary glands by vitamin C administration at 2 and 24 h after an initial treatment using iodine-131 (^131^I) in patients with differentiated thyroid cancer (DTC) and examined the optimal administration time of vitamin C to protect the salivary glands from radiation injury.

**Method:**

The clinical data of patients with differentiated thyroid carcinoma who had been treated with ^131^I in the Department of Nuclear Medicine in Shanxi Bethune Hospital from January 2014 to December 2020 were retrospectively analyzed. The propensity score matching method was adopted to match patients who received the administration of vitamin C at 2 h with those receiving administration at 24 h. A total of 230 pairs/460 patients were enrolled in the study. The chi-squared (*χ*^2^) or Fisher's exact test was used to compare the indicators representing the incidence of salivary gland injury between the two groups.

**Results:**

The incidence of salivary gland injury (17.39%) with acidic substances at 2 h was lower compared with administration at 24 h (26.96%). The incidence of acute salivary gland injury (15.22%) and chronic salivary gland injury (26.09%) in the 24-h group were higher than those in the 2-h group (4.78% and 18.26%, respectively). The differences in the left submandibular gland concentrate index and right submandibular gland concentrate index were statistically significant before and after treatment in both the 2 and the 24-h groups; these functions had been impaired after treatment.

**Conclusions:**

Following treatment with ^131^I, the protective effect of acidic substances administered at 2 and 24 h on the salivary glands were different. The incidence of salivary gland injury in the 2 h acid stimulation group was lower than in the 24 h acid stimulation group. The present study revealed that ^131^I treatment did cause some injury to the salivary glands and that the protective effect of administering vitamin C at 2 and 24 h may be limited. Accordingly, protection against salivary gland injury should be conducted using comprehensive measures.

## Introduction

Supplemented with the oral administration of radioactive iodine (RAI) treatment after surgery for a variety of differentiated thyroid cancers (1, 2), iodine-131 (^131^I) will not only concentrate in residual thyroid tissue and where differentiated thyroid carcinoma metastasizes but also in the salivary glands, lacrimal glands, gastric mucosa, and lactating breasts (3) because these tissue types effect sodium/iodide symporter expression in a similar manner to the thyroid gland (2, 4). Therefore, when ^131^I accumulates in the salivary glands it may cause salivary gland damage.

The most common short-term complication of radiation injury to the salivary glands is acute sialadenitis, which occurs within the first 48 h after radioactive iodine (5) and primarily presents as pain and swelling (6, 7). In addition, long-term complications may occur in some cases; these may manifest as chronic sialadenitis where a persistent dry mouth is a prominent symptom (6, 8, 9).

Currently, sialagogues, such as vitamin C in lemon, are often administered as a preventive measure to minimize absorbed doses of ^131^I in the salivary glands (2, 10, 11). This can increase the flow of saliva and the excretion of radioactive iodine to protect the salivary glands. However, since acidic substances increase the flow rate of saliva, which in turn increases the intake of blood by salivary glands, and finally increases the intake of radioactive iodine (12–14), scholars disagree on the specific intake time of acidic substances. As such, an optimal administration time has not yet been determined in this regard.

This study retrospectively analyzed postoperative patients with differentiated thyroid carcinoma who had been treated with ^131^I in the Department of Nuclear Medicine of Shanxi Bethune Hospital from January 2014 to December 2020. The propensity score matching (PSM) method was used to compare the effects of 2 and 24 h vitamin C administration on salivary gland radiation damage. This study provides a relevant evidence for clinical determination of the optimal administration time of vitamin C.

## Materials and method

### Patients and data collection

A retrospective collection of postoperative patients with differentiated thyroid carcinoma who had been treated with ^131^I in the Department of Nuclear Medicine, Shanxi Bethune Hospital from January 2014 to December 2020 were included in the study. The enrolled patients were treated with ^131^I for the first time. Salivary gland dynamic imaging was performed before treatment to assess basic salivary gland function; 4–6 months after ^131^I treatment, salivary gland dynamic imaging was performed again to evaluate salivary gland function. All radioactive iodine treatments were performed under the stimulation of endogenous thyroid-stimulating hormone (TSH) to achieve a serum TSH level ≥30 mIU/L. The patients had been incorporating a low-iodine diet for at least one month and adopted the same drinking and eating schedule after ^131^I treatment.

The patients were divided into a 2 and 24-h group, respectively, according to the time of starting vitamin C administration after taking ^131^I. The patients in both groups were given 100 mg of vitamin C once every hour (except for while asleep between 23:00 and 6:00) for five consecutive days.

The study's exclusion criteria were as follows: (1) Patients who had a history of salivary gland injury, patients who had received external irradiation therapy for the head and neck, or patients who had a history of connective tissue, such as systemic lupus erythematosus, Sjogren's syndrome, systemic sclerosis and other connective tissue diseases that affect salivary gland function; (2) patients who were taking drugs that affected saliva secretion by the salivary glands, e.g., anticholinergic drugs, antidepressants, and beta-adrenergic blockers; (3) patients who were lost to follow-up. The following clinical patient information was recorded in detail: gender, age, TSH level, number of surgeries, whether Hashimoto's thyroiditis had been combined, clinical stage, metastases, and ^131^I dose.

### Dynamic salivary gland scintigraphy (SGS)

Single-photon emission computed tomography (SPECT) was conducted using the Discovery 670 (General Electric) imaging platform equipped with a low-energy high-resolution collimator. The patient lay on the examination bed with their head slightly tilted back. A dynamic scan acquisition process (using a 64 × 64 matrix, magnification 1.0,30 frames every 60 s, and the photopeak centered at 140 keV with a 20% window width) was initiated simultaneously using a 99mTc-pertechnetate cubital vein (370 MBq) “projectile”. At 20 min, the patient was given vitamin C (200 mg) to stimulate saliva secretion.

According to the vitamin C administration time, the participants were divided into 2 and 24-h groups, respectively. The present research reflects a retrospective study in which there were confounding factors between the two groups; as such, it was impossible to judge whether the difference in clinical results had been due to different vitamin C administration times. Accordingly, the PSM was used for further analysis following 1:1 matching. The matching data variables included gender, age, thyroid stimulating hormone (TSH) level, number of surgeries, whether Hashimoto's thyroiditis had been combined, clinical stage, metastases, and ^131^I dose.

### Diagnostic criteria for salivary gland damage

The clinical symptoms criteria for the present study were salivary gland swelling, as well as swelling and pain with one of the following symptoms: dry/bitter mouth, dysphagia, and a decreased tasting function, judged as salivary gland inflammation.

**Figure 1:**
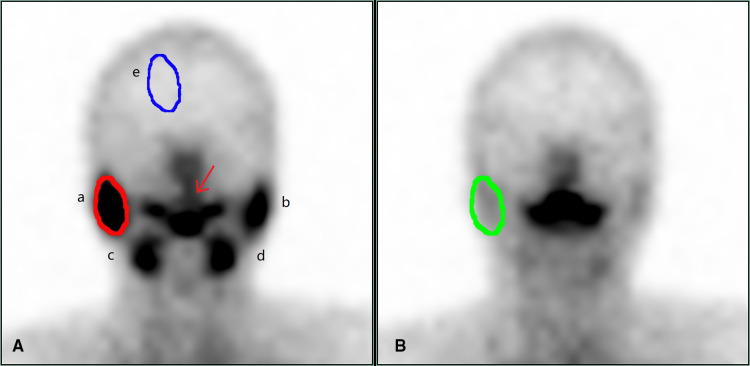
Dynamic salivary gland scintigraphy. (**A**) is the frame with the highest radioactive count uptake by the salivary glands, and (**B**) is the frame with the lowest radioactive counts after the salivary gland secretion. In (**A**), a is the right parotid gland, b is the left parotid gland, c is the right submandibular gland, d is the left submandibular gland, and e is the background, draw the ROI to obtain the radioactivity count, which is (**B**) in the formula. The red arrow is the saliva secreted by the salivary glands. In (**A**), the red circle outlines the right parotid gland, and the ROI is drawn to obtain the radioactive count, which is the highest count concentrated by the right parotid gland, which is the C_max_ in the formula. In (**B**), the green circle outlines the right parotid gland, and the ROI is drawn to obtain the radioactive count, which is the lowest count after the secretion by right parotid gland, that is C_min_ in the formula. Draw b, c, d in turn, and calculate the concentrate index and secretion index of each gland according to the following formulas. Concentrate index (CI) = (C_max_–B)/ B secretion index (SI) = (C_max_–C_min_)/C_max_ × 100%. C_max_ = gland highest radioactive count; C_min_ = the lowest gland radioactivity count after vitamin C stimulation; B = background radioactivity count.

Objective image evaluation was carried out as follows: Image analysis was conducted by two physicians with intermediate or higher professional titles using the double-blind method, and existing relevant clinical reports and clinical data remained unknown. Region of interest technology was used to manually illustrate corresponding areas of the bilateral parotid, submandibular, and background. The time-radioactive curves (TAC) of the bilateral parotid and submandibular glands were computer-generated, corresponding functional parameters were obtained according to the time-radioactive curves, and the average value of salivary gland functional parameters was calculated as statistical data to evaluate the function of each gland. The parameters that reflected the function of the glands were the following: concentrate index (CI) = (C_max_–B)/B; secretion index (SI) = (C_max_–C_min_)/C_max_ × 100% (C_max_ = gland highest radioactive count; C_min_ = the lowest gland radioactivity count after vitamin C stimulation; B = background radioactivity count) (Figure 1).

#### Evaluation method

Based on the evaluation of salivary gland function by Umehara et al. (15) and the scoring method applied in a study conducted by Shizukuishi et al. (16), the intake and secretion functions of the salivary glands were divided into four levels, i.e., normal, mild dysfunction, moderate dysfunction, and severe dysfunction.

#### Evaluation of salivary gland concentrate function

The following results were established for the concentrate by the salivary glands. Normal (C0): concentrate index ≥5; mild functional decline (C1): 3.5 ≤concentrate index <5; moderate functional decline (C2): 2 ≤ concentrate index <3.5; severe functional decline (C3): concentrate index <2.

#### Evaluation of salivary gland secretory function

The following results were established for salivary gland secretion function. Normal secretory function: secretion index ≥40%; mild dysfunction: 20% ≤ secretion index <40%; moderate dysfunction: 10% ≤ secretion index <20%; severe dysfunction: secretion index <10%. The concentrate index and secretion index of the bilateral parotid and submandibular glands before and after treatment with ^131^I were calculated, and changes in the concentrate index and secretion index of each gland before and after treatment with ^131^I were calculated.

#### Evaluation of the changes in intake function

The following results were established for changes in intake function. No change in concentrate index; For mild functional impairment, the concentrate index decreased by one level; for moderate functional impairment, the concentrate index decreased by two levels; for severe functional impairment, the concentrate index decreased by three levels.

#### Evaluation of the changes in secretory function

The following results were established for changes in salivary gland secretory function. For no change, the reduction in secretion index was less than 11%; For mild functional impairment, the secretion index reduction of 11%–20% was observed; for moderate functional impairment, the secretion index reduction of 21%–30% was detected; for severe functional impairment, the secretion index reduction of more than 30% was observed.

The participants received follow-up of the salivary glands up to six months after treatment with ^131^I and whether the salivary glands had been damaged was judged according to clinical symptoms and a functional evaluation. Only when both clinical symptoms and dynamic salivary gland scintigraphy dysfunction existed was a judgment of salivary gland damage made.

### Observation index

#### Clinical symptoms

The incidence of acute and chronic salivary gland damage was observed. Salivary gland damage can be divided into two types according to their time of occurrence. The first of these is acute damage, which occurs primarily within a few days to a month after treatment and chiefly manifests as pain and swelling of the salivary glands. The second type is chronic damage, which primarily occurs one month after treatment with ^131^I and manifests in different ways, including decreased saliva secretion, a decrease in saliva outflow and its overall outflow rate, taste changes, varying degrees of dry mouth, dental caries, severe breath, and salivary adenitis.

Salivary gland damage in dynamic salivary gland scintigraphy: if one of the four salivary glands showed impaired concentrate index or secretion index after ^131^I treatment, dynamic salivary gland scintigraphy was considered abnormal. According to dynamic salivary gland scintigraphy, concentrate index and secretion index of parotid and submandibular glands were recorded, respectively.

### Statistical analysis

The SPSS Statistics 26.0 software program was used for the PSM and data analysis. The propensity value of each patient was calculated according to their variable data, and patients with the closest propensity value were matched 1:1 with a matching tolerance of 0.02. Normally distributed quantitative data were expressed as mean ± standard deviation, and a t-test and variance analysis was used for comparison between groups. Non-normally distributed quantitative data were expressed by M (Q_R_), and a Wilcoxon rank-sum test was used for comparison between the groups; categorical data were expressed in frequency and percentage. The comparison of disorderly classified data between groups employed an *χ*^2^ or Fisher's exact probability test, ordinal data were compared using the rank sum test. The difference was considered statistically significant if *P* < 0.05.

## Results

### Basic clinical data of patients after PSM

Before matching, a total of 872 patients were enrolled, 324 in the 2-h group and 548 in the 24-h group; the age (x ± s: 45.82 ± 11.495 vs. 44.18 ± 12.309, respectively, *t* = 1.993, *P* = 0.047), dose [M(Q_R_): 100(20) mCi vs. 150(50) mCi, respectively, W = 102,004.5, *P* = 0.000], and metastases (*χ*^2 ^= 84.024, *P* = 0.000) of the 2 and 24-h groups showed statistically significant differences. Using PSM, 230 patients in the 2 and 24-h groups were successfully matched. After PSM no statistically significant differences were found in the clinical data between the two patient groups (Table 1).

**Table 1:** Comparison of general data between 2-h group and 24-h group after propensity score matching.

Items	2-h group	24-h group	*P-*value
	230	230	
Gender (male/female)	66/164	67/163	0.918
Age (mean ± SD, years)	45.06 ± 11.553	45.29 ± 11.790	0.836
TSH level (mean ± SD, uIU/ml)	62.79 ± 19.07	61.89 ± 19.46	0.617
Operation frequency (once/ two or more)	186/44	182/48	0.641
Hashimoto’s thyroiditis (no/yes)	182/48	188/42	0.481
Clinical stage (I/II/III/IVA/IVB)	177/35/6/1/11	170/43/8/0/9	0.586
Metastasis			
(no/metastasis with iodine uptake/ metastasis with no iodine uptake)	150/50/30	138/67/25	0.180
^131^I dose (M(Q_R_), mCi)	110 (50)	100 (50)	0.130

After Following PSM matching, no statistically significant differences were found in the clinical data between the two patient groups.

### Comparison of the incidence of salivary gland damage

There was no significant difference in concentrate and secretion functions in the parotid and submandibular glands before treatment with ^131^I between the 2 and the 24-h groups (left parotid gland concentrate, *χ*^2 ^= 3.613, *P* = 0.306; left parotid gland secretory function, *χ*^2 ^= 0.757, *P* = 0.860; right parotid gland concentrate, *χ*^2 ^= 3.984, *P* = 0.263; right parotid gland secretory function, *χ*^2 ^= 1.137, *P* = 0.768; left submandibular gland concentrate, *χ*^2 ^= 2.357, *P* = 0.502; left submandibular gland secretory function, *χ*^2 ^= 1.423, *P* = 0.700; right submandibular gland concentrate, *χ*^2 ^= 6.548, *P* = 0.089; right submandibular gland secretory function, *χ*^2 ^= 1.454, *P* = 0.483). Only when both clinical symptoms and dynamic salivary gland scintigraphy dysfunction were present was a judgement of salivary gland damage confirmed. The incidence of salivary gland damage in the 2-h group was 17.39%, and the incidence of sialadenitis in the 24-h group was 26.96%; the incidence of salivary gland injury in the 2-h group was lower than that in the 24-h group; there was a statistical difference between the two groups (Table 2).

**Table 2:** Comparison of salivary gland damage between 2-h group and 24-h group.

Groups	No salivary gland damage	Salivary gland damage	Total
2-h	190 (82.61%)	40 (17.39%)	230
24-h	168 (73.04%)	62 (26.96%)	230
Total	358	102	460

In Table 2, only when both clinical symptoms and dynamic salivary gland scintigraphy images dysfunction were present was a diagnostic salivary gland damage. The incidence of salivary gland damage in the 2-h group was 17.39%, and the incidence of sialadenitis in the 24-h group was 26.96%; the incidence of salivary gland injury in the 2-h group was lower than that in the 24-h group; there was a statistical difference between the two groups.

*χ*^2 ^= 6.097, *P* = 0.014.

### Comparison of clinical symptoms of salivary gland damage

The incidence of clinical symptoms linked to salivary gland damage was 20% in the 2-h group and 37.39% in the 24-h group, indicating the 2-h group was lower than the 24-h group, and the difference between the two groups was statistically significant (Table 3). The incidence of acute salivary gland damage in the 2-h group was 11/230 (4.78%) compared with 35/230 (15.22%) in the 24-h group. The 2-h group was lower than the 24-h group and there was a statistical difference between the two groups (*χ*^2 ^= 13.913, *P* < 0.001) (Table 4). The incidence of chronic salivary gland damage in the 2-h group was 42/230 (18.26%) and 60/230 (26.09%) in the 24-h group. The 2-h group was lower than the 24-h group and there was a statistical difference between the two groups (*χ*^2 ^= 4.081, *P* = 0.043) (Table 5). In patients with acute salivary gland damage, the incidence of chronic salivary gland damage in the 2 and 24-h groups was 7/11 (63.64%) and 9/35 (25.71%), respectively, and there was no statistical difference between the two groups (*χ*^2 ^= 3.766, *P* = 0.052). In patients with chronic salivary gland damage, the incidence of acute salivary gland damage in the 2 and 24-h groups was 7/42 (16.67%) and 9/60 (15%), respectively, and there was no statistical difference between the two groups (*χ*^2 ^= 0.052, *P* = 0.820).

**Table 3:** Comparison of clinical symptoms of salivary gland damage between 2-h group and 24-h group.

Groups	No clinical symptoms	Presence of clinical symptoms	Total
2-h	184 (80.00%)	46 (20.00%)	230
24-h	144 (62.61%)	86 (37.39%)	230
Total	328	132	460

In Table 3, whether dynamic salivary gland scintigraphy images are abnormal or not, the clinical symptoms criteria for the present study were salivary gland swelling, as well as swelling and pain with one of the following symptoms: dry/bitter mouth, dysphagia, and a decreased tasting function, judged as salivary gland inflammation. The incidence of clinical symptoms linked to salivary gland damage was 20% in the 2-h group and 37.39% in the 24-h group, indicating the 2-h group was lower than the 24-h group, and the difference between the two groups was statistically significant.

*χ*^2 ^= 16.999, *P* < 0.001.

**Table 4:** Comparison of acute salivary gland damage between 2-h group and 24-h group.

Groups	No acute salivary gland damage	Presence of acute salivary gland damage	Total
2-h	219 (95.22%)	11 (4.78%)	230
24-h	195 (84.78%)	35 (15.22%)	230
Total	414	46	460

In Table 4, the incidence of acute salivary gland damage was compared according to the clinical symptoms of the patients. The acute salivary gland damage occurs primarily within a few days to a month after ^131^I treatment and chiefly manifests as pain and swelling of the salivary glands. The incidence of acute salivary gland damage in the 2-h group was 11/230 (4.78%) compared with 35/230 (15.22%) in the 24-h group. The 2-h group was lower than the 24-h group and there was a statistical difference between the two groups.

*χ*^2 ^= 13.913, *P* < 0.001.

**Table 5:** Comparison of chronic salivary gland damage between 2-h group and 24-h group.

Groups	No chronic salivary gland damage	Presence of chronic salivary gland damage	Total
2-h	188 (81.74%)	42 (18.26%)	230
24-h	170 (73.91%)	60 (26.09%)	230
Total	358	102	460

In Table 5, the incidence of chronic salivary gland damage was compared according to the clinical symptoms of the patients. The chronic salivary gland damage occurs primarily one month after treatment with ^131^I and manifests in different ways, including decreased saliva secretion, a decrease in saliva outflow and its overall outflow rate, taste changes, varying degrees of dry mouth, dental caries, severe breath, and salivary adenitis. The incidence of chronic salivary gland damage in the 2-h group was 42/230 (18.26%) and 60/230 (26.09%) in the 24-h group. The 2-h group was lower than the 24-h group and there was a statistical difference between the two groups.

*χ*^2 ^= 4.081, *P* = 0.043.

### Comparison of salivary gland damage in salivary gland scintigraphy

**Figure 2:**
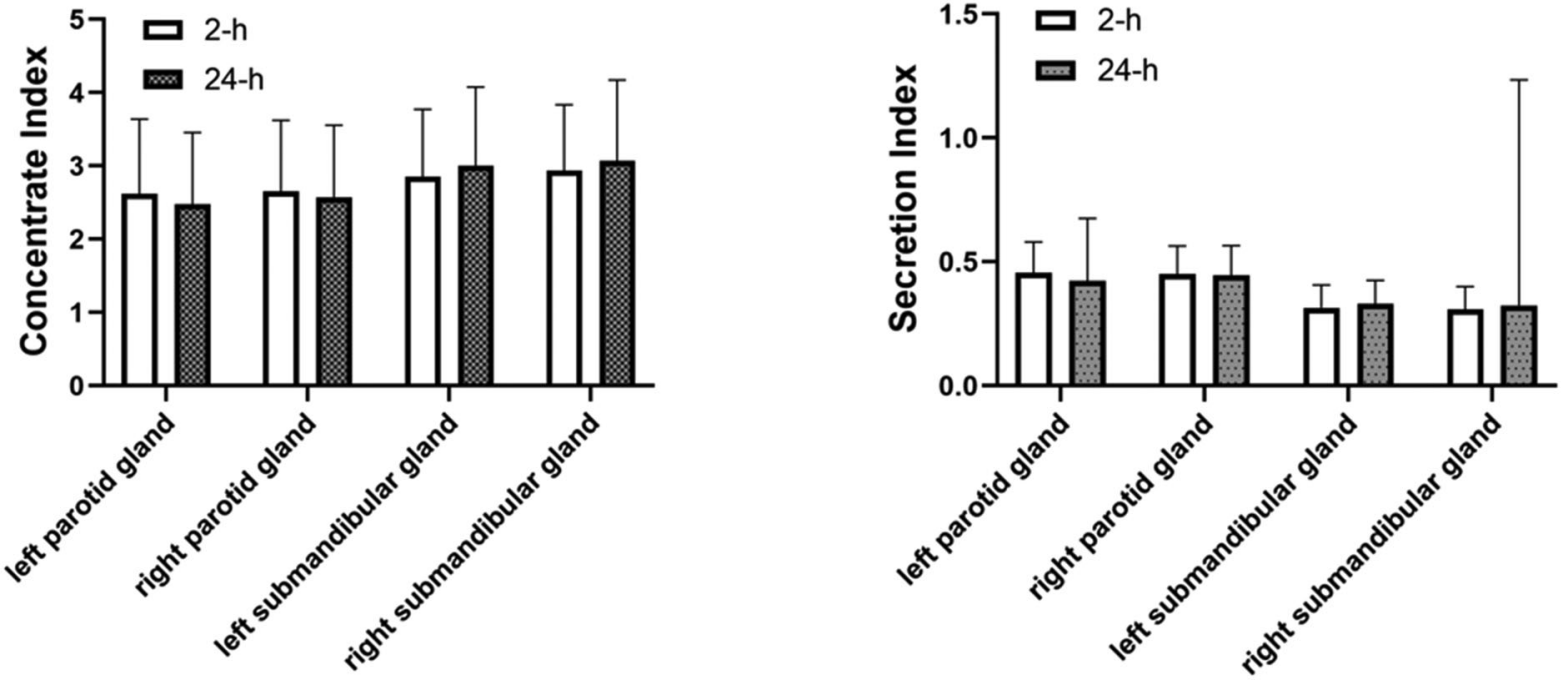
Comparison of concentrate index and secretion index in the parotid and in the parotid and submandibular glands between 2-h group and 24-h group. There was no significant difference in the concentrate and secretion functions in the parotid and submandibular glands after treatment with ^131^I between the 2 and the 24-h group.

In terms of functional impairment in the presence of dynamic salivary gland scintigraphy only, the incidence of salivary gland damage was 70.87% in the 2-h group and 68.26% in the 24-h group; there was no statistical difference between the two groups (Table 6) The parotid gland's functional impairment rate in the 2-h group was 113/230 (49.13%) and 111/230 (48.26%) in the 24-h group. There was no statistical difference between the two groups. The submandibular gland functional impairment rate in the 2-h group was 132/230 (57.39%) and 113/230 (49.13%) in the 24-h group. There was no statistical difference between the two groups. There was no significant difference in the concentrate and secretion functions in the parotid and submandibular glands after treatment with ^131^I between the 2 and the 24-h groups (Figure 2).

**Table 6:** Comparison of functional impairment between 2-h group and 24-h group.

Groups	No functional impairment	Functional impairment	Total
2-h	67 (29.13%)	163 (70.87%)	230
24-h	73 (31.74%)	157 (68.26%)	230
Total	140	320	460

In Table 6, objective dynamic salivary gland scintigraphy image indexes were used to evaluate. If one concentrate index or secretion index of salivary gland was damaged, it was diagnosed as abnormal dynamic salivary gland scintigraphy and damaged salivary gland. In terms of functional impairment in the presence of dynamic salivary gland scintigraphy only, the incidence of salivary gland damage was 70.87% in the 2-h group and 68.26% in the 24-h group; there was no statistical difference between the two groups.

*χ*^2 ^= 0.370, *P* = 0.543.

In patients with clinical salivary gland damage symptoms, the incidence of abnormal dynamic salivary gland scintigraphy in the 2 and 24-h groups was 40/46 (86.96%) and 62/86 (72.09%), respectively, and there was no statistical difference between the two groups (*χ*^2 ^= 3.770, *P* = 0.052). In patients with abnormal dynamic salivary gland scintigraphy, the incidence of salivary gland damage with clinical symptoms in the 2 and 24-h groups was 40/163 (24.54%) and 62/157 (39.49%), respectively. The 2-h group was lower than the 24-h group and there was a statistical difference between the two groups (*χ*^2 ^= 8.232, *P* = 0.004).

### Comparison of salivary gland function before and after ^131^I treatment

**Figure 3:**
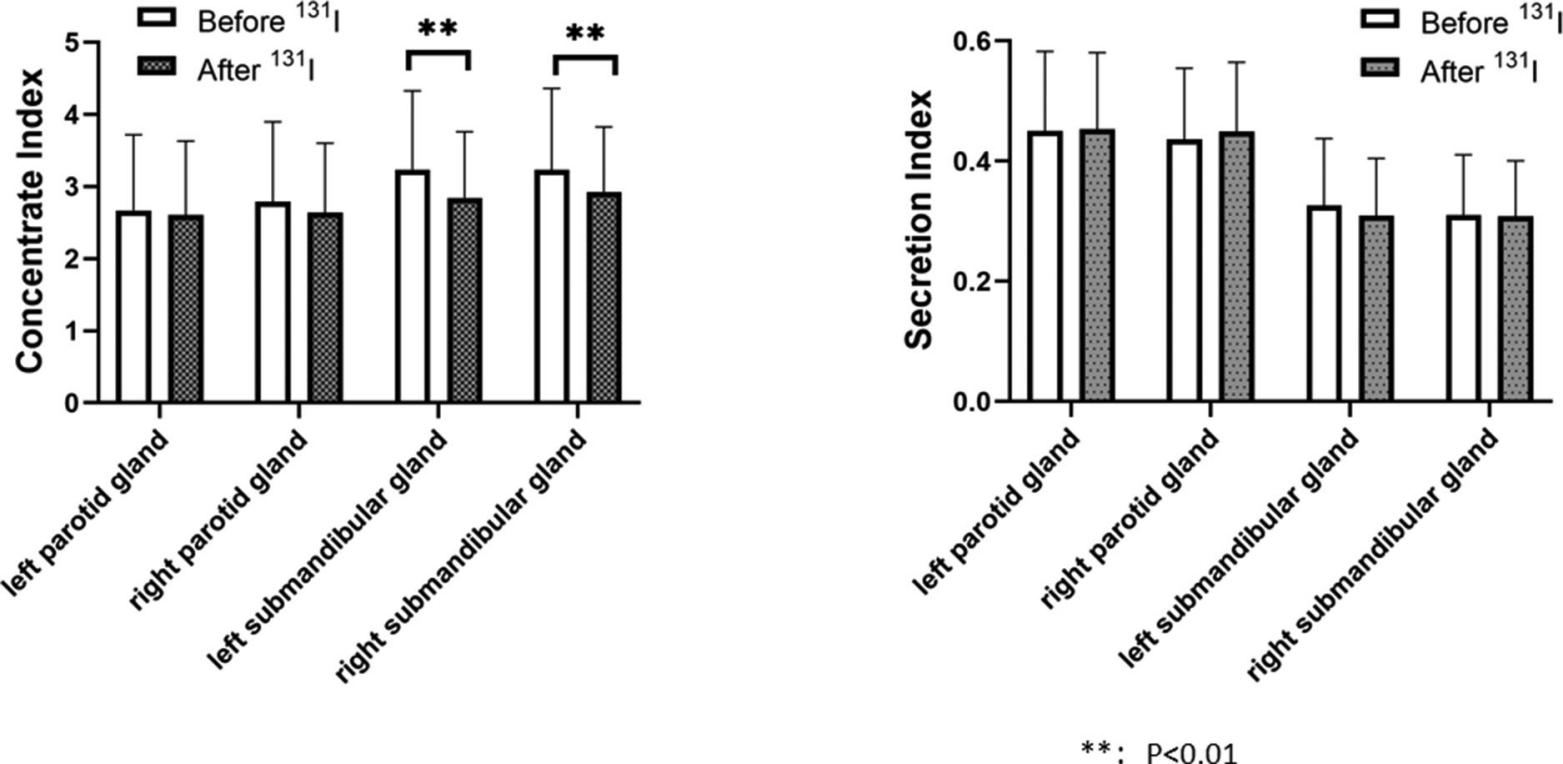
Comparison of concentrate index and secretion index in the parotid and in the parotid and submandibular glands before and after ^131^I treatment in 2-h group. The left submandibular gland concentration index and right submandibular gland concentration index after ^131^I treatment in the 2 h group (2.843 ± 0.916, 2.929 ± 0.898)were lower than those before ^131^I treatment (3.234 ± 1.093, 3.233 ± 1.129), and the changes before and after treatment were statistically different (*P* = 0.001, *P* = 0.002). There was no significant difference in concentration index of the left parotid gland before and after treatment (2.670 ± 1.048, 2.605 ± 1.028, *P* = 0.505), no significant difference in secretion index of the left parotid gland before and after treatment (0.451 ± 0.132, 0.454 ± 0.127, *P* = 0.772), no significant difference in concentration index of the right parotid gland before and after treatment (2.789 ± 1.107, 2.639 ± 0.965, *P* = 0.122), no significant difference in secretion index of the right parotid gland before and after treatment (0.437 ± 0.118, 0.450 ± 0.115, *P* = 0.236), no significant difference in secretion index of the left submandibular gland before and after treatment (0.327 ± 0.111, 0.310 ± 0.095, *P* = 0.080), no significant difference in secretion index of the right submandibular gland before and after treatment (0.311 ± 0.100, 0.309 ± 0.092, *P* = 0.867).

**Figure 4:**
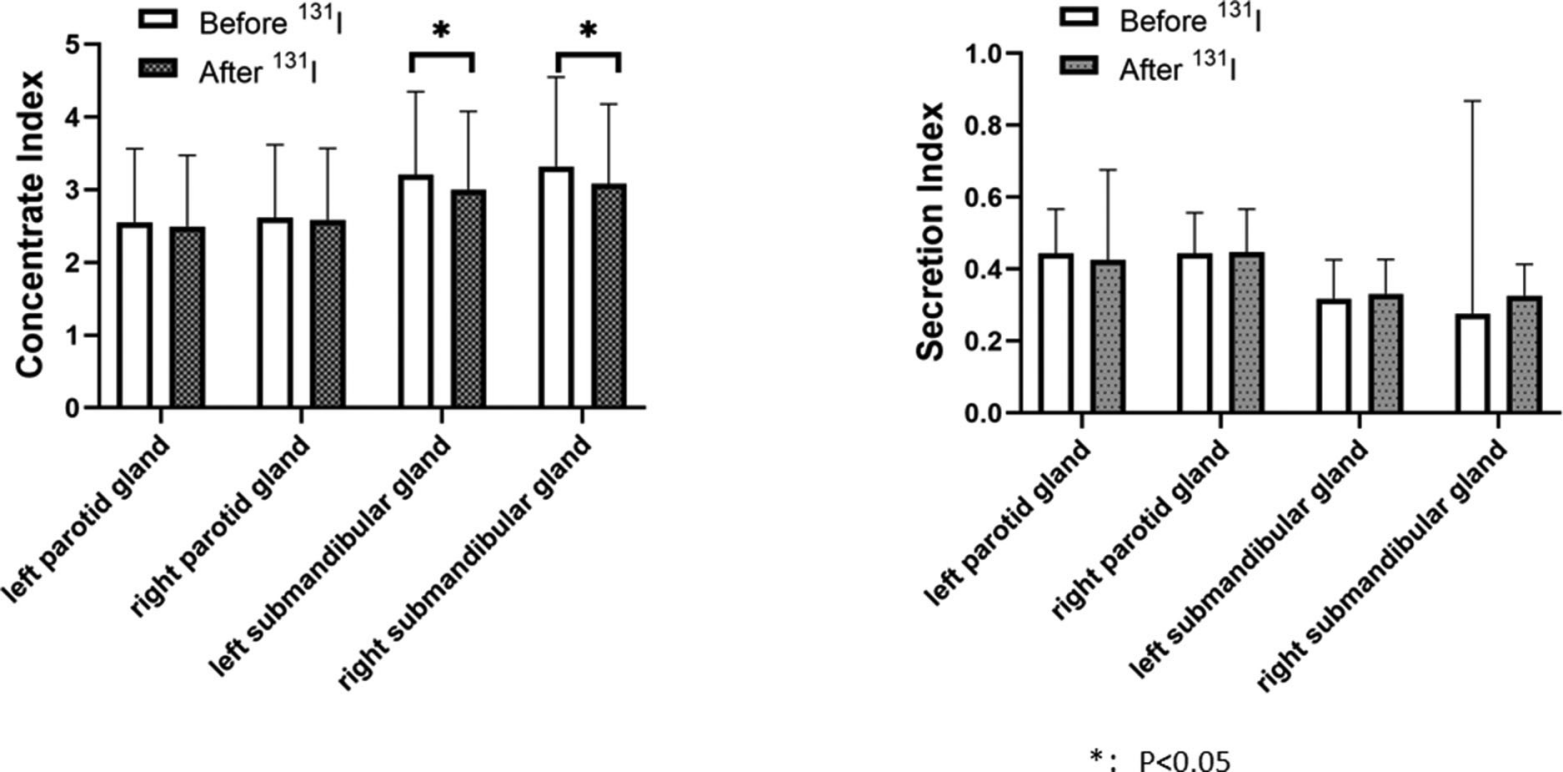
Comparison of concentrate index and secretion index in the parotid and in the parotid and submandibular glands before and after ^131^I treatment in 24-h group. The concentration index of the left submandibular gland and the concentration index of the right submandibular gland in the 24 h group after ^131^I treatment (3.000 ± 1.075, 3.082 ± 1.097) were lower than those before ^131^I treatment (3.205 ± 1.142, 3.318 ± 1.229), and the changes before and after treatment were statistically different (*P* = 0.048, *P* = 0.030). There was no significant difference in concentration index of the left parotid gland before and after treatment (2.552 ± 1.011, 2.493 ± 0.979, *P* = 0.526), no significant difference in secretion index of the left parotid gland before and after treatment (0.444 ± 0.123, 0.426 ± 0.250, *P* = 0.324), no significant difference in concentration index of the right parotid gland before and after treatment (2.613 ± 1.004, 2.586 ± 0.982, *P* = 0.087), no significant difference in secretion index of the right parotid gland before and after treatment (0.444 ± 0.113, 0.447 ± 0.120, *P* = 0.753), no significant difference in secretion index of the left submandibular gland before and after treatment (0.318 ± 0.108, 0.331 ± 0.096, *P* = 0.150), no significant difference in secretion index of the right submandibular gland before and after treatment (0.276 ± 0.592, 0.326 ± 0.088, *P* = 0.210).

The left submandibular gland concentration index and right submandibular gland concentration index after ^131^I treatment in the 2 h group (2.843 ± 0.916, 2.929 ± 0.898)were lower than those before ^131^I treatment (3.234 ± 1.093, 3.233 ± 1.129), and the changes before and after treatment were statistically different (*P* = 0.001, *P* = 0.002), indicating that the salivary gland function after treatment was lower than before treatment (Figure 3). The concentration index of the left submandibular gland and the concentration index of the right submandibular gland in the 24 h group after ^131^I treatment (3.000 ± 1.075, 3.082 ± 1.097) were lower than those before ^131^I treatment (3.205 ± 1.142, 3.318 ± 1.229), and the changes before and after treatment were statistically different (*P* = 0.048, *P* = 0.030), indicating that the salivary gland function after treatment was lower than before treatment (Figure 4).

## Discussion

Radioactive iodine therapy is an accepted treatment for differentiated thyroid carcinoma with rare serious side effects. Among the reported adverse reactions, symptoms of salivary gland inflammation were described relatively frequently, with the incidence ranging from 18.7% to 64.7% (17–19). Radioactive iodine induced sialadenitis (RAIS) is an inflammatory lesion of the salivary gland(20) that was first reported by Rigler and Scanlon (21), and was recently included in a new classification of clinical stomatology (22). Radioactive iodine induced sialadenitis have distinct imaging features in various imaging examinations (23–24). However, dynamic salivary gland scintigraphy is the first choice for follow-up of salivary glands after radioiodine treatment because it is functional imaging and is very sensitive to functional changes (19, 25, 26). The clinical manifestations of salivary gland injury include swelling, pain, and pressure in the parotid area, atrophy of parotid gland, and even atresia of the ductal opening, as well as decreased salivary secretion and thickening and no obvious salivary secretion in some glands. Patients developed dry mouth with taste disturbances or candida infection. Judging salivary gland injury may be too subjective if ruled on by clinical symptoms only. In the case of judging by imaging only, many patients with objective evidence of salivary gland dysfunction do not complain of the symptoms associated with salivary gland dysfunction; therefore, some functional impairment is not sufficient for indicating clinical significance. The literature posits that at least a 50% reduction in dynamic salivary gland scintigraphy was required to reflect clinical consequences (27). In addition, individual differences were observed in the subjective sensory thresholds of patients. Accordingly, the combination of the two is more scientifically reasonable and comprehensive.

Following treatment with^131^I, post-operative patients with differentiated thyroid carcinoma took acidic substances (vitamin C, found in, e.g., lemon) to protect the salivary glands. Although salivary gland cells express sodium iodide symporter for taking up ^131^I, but salivary glands do not have thyroid oxidase in cells, so the residence time of ^131^l in salivary glands is relatively short. The salivary glands take up the most^131^l within 1–2 h after taking ^131^l, and the absorbed dose of salivary glands within 24 h is almost equal to the total absorbed dose of salivary glands (28). At present, vitamin C acid stimulation is often used in clinical practice to reduce salivary gland damage. For the application time of acid stimulation, 2 and 24 h are mostly used. Therefore, a retrospective review on the protective effect of 2 and 24-h administration of vitamin C on the salivary glands was conducted in the present study. Despite its retrospective nature, the research employed propensity matching for eight factors and found no statistical difference between the two groups after matching. Moreover, prior to treatment with^131^I, the difference in salivary function was not statistically significant between the two groups.

When compared under the same conditions, the difference in salivary gland injury incidence was found to be statistically significant between the two groups, with a lower incidence of gland injury with acid administration at 2 h (17.39%) compared with those in the 24-h group (26.96%). Our results are inconsistent with Nakada K's study (14), which showed the incidence of salivary gland injury and oral dryness caused by acid stimulation within 1 h after treatment with ^131^I was significantly higher than in patients with acid stimulation at 24 h; the authors concluded that acid stimulation should only be initiated 24 h after treatment with ^131^I, as early administration not only promoted dynamic salivary gland scintigraphy but also increased blood flow to the gland, and when the effect of promoting increased blood flow to the gland was greater than that of stimulating gland secretion, the acid stimulation could aggravate a salivary gland injury. This was consistent with the findings of Jentzen et al. (29) who conducted a ^124^I positron emission tomography (PET/CT) imaging study and found that the early application of acid stimulation aggravated radiological injury to the salivary glands. However, the data provided by other research failed to support these results, where it is suggested that immediate and repeated dosing may be the best approach (30). Van Nostrand et al. found that repeated administration of acid preparations reduced the radiation-absorbed dose to the parotid gland by 37%–45%, an outcome that supports the continued administration of acid preparations during the day and intermittent application at night (31).

In our study, we found that the incidence of salivary gland injury at 2 h was lower than at 24 h. A reason for this may have been that there could be a time point after treatment with ^131^I. At this time point, the effect of acid stimulation on increasing salivary gland secretion is greater than that on increasing blood perfusion, which could play a role in the protection of salivary glands. This point may be between 2 and 24 h, and the acid stimulation in these patients every hour may cover this possible enable point. Kulkarni et al. (32) conducted a prospective study of ^123^I-scanning in patients, suggesting that the administration of acid preparations should be immediately initiated.

The symptoms of salivary gland injury after iodine treatment were compared between two groups in the current study. The results indicated that 20% of patients in the 2-h group showed symptoms of salivary gland injury while 37.39% of the patients in the 24-h group had symptoms of salivary gland injury. The difference in incidence was statistically significant between the two groups. These results were consistent with other studies in which the incidence of subjective sialadenitis was reported to be between 20% and 35% after treatment with ^131^I (6, 33–36). The differences in the incidence of acute and chronic salivary gland injuries were also compared between the two symptomatic patient groups. The results revealed that the incidence of acute salivary gland injury (15.22%) and chronic salivary gland injury (26.09%) in the 24-h group was higher than those in the 2-h group (4.78%) and 24-h group (18.26%), respectively. Existing literature reported an incidence ranging from 10% to 41% for acute sialadenitis (25, 36–38) and from 5% to 43% for chronic sialadenitis. The results of the present study were consistent with the above studies. The literature (39) noted that in patients with a history of acute sialadenitis, the incidence of chronic salivary gland dysfunction was higher. However, the present study found that patients with acute sialadenitis in both the 2-h group and the 24-h group had a low probability of subsequent chronic sialadenitis, 16.67% in the 2 h group and 15% in the 24 h group. The reason for this phenomenon may be that we reduced the incidence of chronic salivary adenitis by combining salivary gland massage, clearing heat and detoxification, anti-inflammatory and detumescence treatment after acute symptoms. Therefore, it may be necessary to prevent not only acute but also chronic injuries.

In terms of whether acid stimulation may be beneficial for protecting the salivary glands, salivary gland functioning was also compared before and after treatment. Both in the 2 and 24-h groups, the differences in the concentrate and secretion functions of the parotid and submandibular glands were being impaired after treatment. Existing studies have shown that the most commonly adopted radioactive iodine treatment, with an activity of 3.7 GBq, did not stimulate the salivary glands and had no significant influence on salivary gland function (40). Wu et al. (25) did not observe any significant decrease in parotid or submandibular salivary gland function in 78 patients with an activity of 5.55 GBq. However, Maruoka et al. (41) observed a statistically significant decrease in the parotid concentrate and fractional excretion in 193 patients where the radioactive iodine doses ranged from 3.3 to 9.5 GBq. The median dose of ^131^I in the present study was 4.44 GBq. Possible reasons for the inconsistent findings is the administration of acid preparations and the methods of application in each study may serve as confounding factors. Because the guidelines recommend acid stimulation to protect the salivary glands, for humanitarian reasons, we did not have a control group that did not take vitamin C. This study shows that the treatment of ^131^I has a certain damage to the salivary glands. The protection of salivary glands with 2-h and 24-h acid stimulation may be limited, and neither prevents the occurrence of salivary gland damage. The protection of salivary gland damage should be comprehensively prevented.

Despite being retrospective in nature, the novelty of the present study was its use of propensity matching to achieve comparability between two groups of patients. Additionally, the clinical symptoms were combined with imaging functions as indicators for a more comprehensive and scientific evaluation. The study also included some limitations as follows: (1) Only patients who received treatment with ^131^I for differentiated thyroid carcinoma for the first time were enrolled and those with differentiated thyroid carcinoma who had received multiple ^131^I irradiations were not included; (2) In the present study, salivary gland scintigraphy was conducted for patients six months after treatment with ^131^I, and no subsequent salivary gland scintigraphy was conducted. (3) There is no deep research on the mechanism of salivary gland damage rate in the 2 h group being lower than that in the 24 h group, which will be the direction our team will explore.

## Conclusions

1. After undergoing treatment with ^131^I, the protective effect of acidic substances administered at 2 and 24 h on the salivary glands was different. The incidence of salivary gland injury in the 2-h acid stimulation group was lower than in the 24-h acid stimulation group.
2. The present study revealed that treatment with ^131^I caused some injury to the salivary glands, that protection of the salivary gland by the administration of acidic substances at 2 and 24 h may be limited, and that the protection of salivary gland injury should be conducted using comprehensive measures.

## Data Availability

The raw data supporting the conclusions of this article will be made available by the authors, without undue reservation.
